# A pilot case crossover study of the use of padded headgear in junior Australian football

**DOI:** 10.2217/cnc-2022-0005

**Published:** 2023-01-17

**Authors:** Catherine Willmott, Jonathan Reyes, Jack V K Nguyen, Andrew McIntosh, Jennifer Makovec-Knight, Michael Makdissi, Patrick Clifton, Peter Harcourt, Biswadev Mitra

**Affiliations:** 1 Turner Institute for Brain & Mental Health, School of Psychological Sciences, Monash University, Melbourne, Australia; 2 Monash-Epworth Rehabilitation Research Centre, Epworth Hospital, Melbourne, Australia; 3 McIntosh Consultancy and Research, Sydney, Australia; 4 Monash University Accident Research Centre, Monash University, Melbourne, Australia; 5 School of Engineering, Edith Cowan University, Joondalup, Australia; 6 Florey Institute of Neuroscience & Mental Health, Austin Campus, Melbourne Brain Centre, Melbourne, Australia; 7 Olympic Park Sports Medicine Centre, Melbourne, Australia; 8 Australian Football League, Melbourne, Australia; 9 National Trauma Research Institute, The Alfred Hospital, Melbourne, Australia; 10 Emergency & Trauma Centre, The Alfred Hospital, Melbourne, Australia; 11 Department of Epidemiology & Preventive Medicine, Monash University, Melbourne, Australia

**Keywords:** behavior, headgear, injury surveillance, mild traumatic brain injury, sport-related concussion, youth football

## Abstract

**Aim:**

To explore soft-shell padded headgear (HG) use, player behavior and injuries associated with HG in junior Australian football.

**Methods:**

Prospective case-crossover with head impact measurement, injury surveillance and video review.

**Results:**

40 players (mean age: 12.43 years, standard deviation: 1.36) across 15 matches were observed. Frequency of head/neck (p = 0.916) or body (p = 0.883) contact events, and match incidents were similar between HG and no HG conditions. Without HG, females had higher frequency of body contacts compared with males (p = 0.015). Males sustained more body contacts with HG than without HG (p = 0.013).

**Conclusion:**

Use of HG in junior football was not associated with injury or head contact rate. Associations between HG use and body contact may differ across sexes. (ID: ACTRN12619001165178).

Soft-shell padded headgear (HG) is inconsistently mandated as a preventive strategy for sports-related concussion (SRC) in junior and youth Australian football, despite limited evidence [1]. Major limitations of available evidence for HG use in youth Australian football are generalizability from studies conducted in rugby [2,3] and soccer [4,5], and the predominance of all male cohorts, particularly important given the increasing female participation rates in Australian football [6]. Potential associations between HG use and SRC are weak, as are associations of other injury rates and HG use [7,8]. One recent study in junior Australian football found that HG use was not associated with reduced risk of SRC or other injuries [9].

Although prior biomechanical evidence indicates that commercially available HG are unlikely to prevent SRC in any player cohort [7,10], it has been suggested that one reason for the absence of benefit in observational studies is the effect of HG on player behaviors [11]. Synonymous with ‘risk homeostasis theory’, risk compensation was first posited by Peltzman who suggested that increases in traffic accidents were a response to the introduction of safety regulations, wherein greater perceived safety was associated with behavior change in a way that returns to the original level of desired risk [12,13]. Risk compensation theory suggests that either the full benefits of HG, or any safety intervention, will not be observed and/or there will be an increased overall injury incidence associated with wearing HG as a result of behavior change. Reviews on the subject of risk compensation in sports that employ hard shell helmets have concluded that the use of safety helmets does not appear to increase risk behaviors when compared with non-helmeted participants in skiing and snowboarding [14] or cycling [15]. The latter supports the views of Pless *et al.* who argued that if risk compensation is true, more severe injuries would be expected in helmeted bicycle riders because of increased risk-taking behaviors [16]. In contact sport, the risk compensation theory posits that players feel that they can tackle harder and are more confident when wearing HG [17,18], and therefore their behavior on the field is altered, which may result in more aggressive play with less concern for safety [19]. This contention is supported to some extent by survey findings in which young players endorse beliefs that HG makes them feel safer and enables them to play harder [17,20]. Whether or not general risk taking and sensation seeking modifies this behavior remains to be explored [21].

Documentation and analysis of injury events in sport is commonly explored using post-match video coding of such events from match day footage [22]. Identification of contact events across match situations as identified on subsequent video review across HG conditions represents a reliable way to determine whether HG use is associated with a change in player behavior. Such changes in behavior, if evident, may represent risk compensation associated with HG use [14].

To date there have been no randomised controlled trials of HG in junior Australian football, likely due to multiple reasons. These include the lack of evidence demonstrating efficacy for a reduction in SRC incidence in other sporting codes; the considerable methodological difficulties associated with injury surveillance in such cohorts including substantial resource requirements and voluntary nature of those who run such competitions [23] and in Australian football the fact that very few players wore HG prior to the past ten years or so. Despite “insufficient evidence to make a recommendation for the use of helmets for the prevention of concussion in Australian Football” [p.13; 24], a substantial number of community clubs currently mandate HG at the junior and/or youth levels of the game [1]. In some instances, HG is provided by the club, whereas other clubs require parents to supply the HG.

There are currently no mandated product standards for HG in Australia and HG sold in Australia either meets no product standard or complies with World Rugby's HG regulation. Laboratory evidence has demonstrated consistently the inadequate impact attenuation performance of commercially available HG models, including those complying with World Rugby's regulation [7]. While rotational forces (angular acceleration and angular momentum), which play an integral part in the mechanisms of concussion and are a critical component of the evaluation of head impact mitigation properties of HG, are integrated into some HG standards, these forces are not always evaluated in studies which are restricted to linear acceleration forces only.

The increasing availability of wearable sensor technology provides an opportunity to quantify head acceleration events (HAEs) in contact sports [25,26], and this has recently been trialled in elite Australian football [27]. Consistent with previous studies quantifying contact exposure in soccer and lacrosse [28,29], control measures including video verification and minimum acceleration thresholds should be implemented to control for possible device errors (i.e., detection of head acceleration due to non-contact events such as jumping) [27]. The use of accelerometers has the potential to characterize the nature of head impacts sustained in junior and youth Australian football, however, no study to date has examined differences in the frequency and magnitude of HAE among youth Australian football players across HG and no HG conditions.

The primary aim of this pilot trial was to determine feasibility in a case-crossover pilot study of HG use in junior Australian football. The secondary aims were to evaluate player behavior across HG and no HG conditions (measured by frequency of head/neck and body contact events as identified on video coding) in male and female players. quantify contact events (frequency and magnitude) by use of an accelerometer device, investigate the association between self-reported risk taking propensity and player behavior across HG conditions and document match incidents and injuries sustained.

## Methods

### Setting

In Australian football, players are required to dispose of the football by handballing or kicking the football. ‘Marking contests’ occur wherein players attempt to ‘mark’ (i.e., catch) a football without the opposition or ground contacting the football first. The football may be contested on the ground wherein any player who gains possession of the football can be tackled by the opposition. In ground contest scenarios, players can ‘shepherd’ the ball carrier by using their own body to ‘bump’ or ‘block’ the opposition from tackling the ball carrier. This sport therefore has multiple sources of permissible body contact at the adult level of the game. While there is no mandated wear of protective equipment for players, the AFL strongly encourage the use of mouthguards to prevent dental injuries [30]. Rule modifications, however, are in place at the junior level of the sport, wherein tackling – for example – is not allowed (e.g., U8 players), or is modified (e.g., U9 to U10 players) until progressing to the adult laws of the game [31]. Hard-shell helmets are not permitted in Australian football, but HG and other forms of personal protective equipment (e.g., mouth guards) are either permitted or mandated in selected competitions [30].

### Participants

One junior under 12 years (U12) male Division 2 and three youth (one under 14 years (U14) male Division 1 and two U14 female Division 1 and Division 2) football teams of the Southern Metro Junior Football League competing in 2019 initially agreed to participate in the study. These age groups were selected in order to ensure some equivalence in the football by-laws across the sexes (e.g., full tackling is permitted for teams of U14 females and above, and for U12 males and above).

### Design

Feasibility in this case crossover pilot study was measured by player assent and compliance with HG use. Inclusion criterion was being a listed player in the targeted teams outlined above and there were no exclusion criteria. All participating players and parents of players provided written informed assent and consent respectively in this case-crossover pilot study design study approved by the Monash University Human Research Ethics Committee (Project Number: 11254). The trial was retrospectively registered during data collection on 20/08/2019 (ID: ACTRN12619001165178).

**Figure 1.**
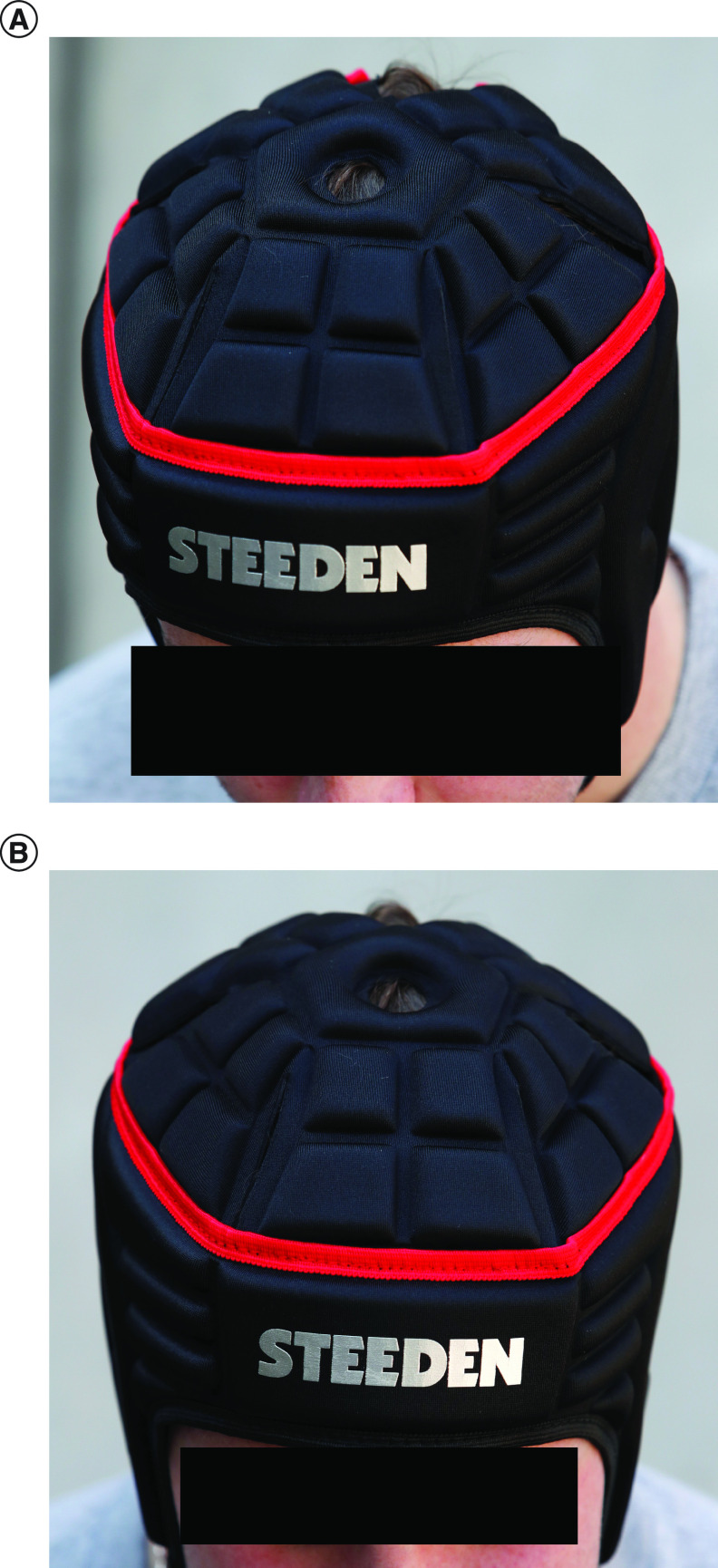
Soft-padded headgear utilised in this study. **(A)** Front–side view. **(B)** Front view.

On match day, all participating players were provided with, and wore, HG (Steeden Super Lite manufactured by Steeden^®^) for the same half of the match, and no HG for the other half (Figure 1). The half of the match (first or second) with the allocated HG was randomised across the 15 matches assessed with use of a computerised random number generator. The Steeden model was chosen due to its popularity and availability [1]. Player compliance rates with wearing HG were documented. Opponent teams across each of these 15 matches were from other non-participating clubs and were therefore not participants in the trial.

### Data collection & variables

Data collection occurred across five matches (out of a season of ~10 matches) for each of the three Australian football participating teams (i.e., 15 matches total). Research personnel conducted all demographic and risk propensity data collection prior to match day. Prior to commencement of the match, X-Patch^®^ (purchased in 2015 from X2 Biosystems Inc., WA) application was undertaken by research personnel, and HG application occurred pre-match or at half-time as per the randomisation schedule. Injury surveillance was carried out across the duration of the match, and each of the 15 matches were staffed by three research personnel to optimize HG application and injury surveillance.

#### Demographics & risk propensity

Demographic data collection included age, weight and height. Risk propensity was screened using a modified version of the Risk Propensity Scale (RPS; Cronbach's α = .77) [32]. In the modified version utilised within the present study for administration in a paediatric cohort, the RPS score was calculated across four items rated on a scale of 1 (*strongly disagree*) to 5 (*strongly agree*), with scores reversed for item 1 and 4 in order to produce a total RPS score (range 4 to 20) with higher scores indicating greater risk-taking propensity [32].

#### Accelerometer data

X-Patch^®^ accelerometer devices were placed behind the ear over the mastoid of participants using double-sided adhesives and further secured with sports tape (manufactured by BodyPlus^®^) across the device, with a counterbalance of application to the left and right mastoid across the five match participations. X-Patch^®^ devices were worn for the entire match.

The X-Patch^®^ contains a triaxial linear accelerometer and triaxial gyroscope sensor with only peak linear acceleration (PLA) recommended for use. McIntosh *et al.* conducted laboratory-based and *in-vivo* analysis of the X-Patch^®^ demonstrating greater reliability for PLA (up to 17% variance from gold standard reference values obtained with a laboratory-based headform assessment) in comparison to peak rotational acceleration (PRA) values (up to 246% variance) consistent with review by Wu *et al.* [33,34].

The X-Patch^®^ continuously samples at a frequency of 1–kHz and is triggered to record 100–ms of time-stamped data for any acceleration event with a PLA exceeding 10 *g* and cannot differentiate between a direct head impact or an impact to another body location resulting in an inertial loading of the head [28,34,35]. A PLA threshold ≥30 *g* was also examined on the basis that HAEs with PLA <30 *g* alone may be confounded by non-contact sporting or everyday activity, such as jumping, running at high speeds then rapidly stopping, or landing [28,36–38].

The Impact Monitoring System (IMS; X2 Biosystems) proprietary software was used to obtain a time-stamped dataset of multiple PLA values per accelerometer device per player. The IMS in-built algorithm to identify false positive PLAs was disabled due to the lack of reliability of this feature and all PLAs were included for analysis [28,39].

#### Video review

In order to detect differences in player behavior across HG and no HG conditions, all 15 matches were professionally video recorded and reviewed from three separate angles of the playing surface in order to optimize the field of view. The video review was conducted by two neuropsychology trainees (JR and JN) using an adapted version of a previously developed protocol [27] and inter-rater reliability was assessed. Player behavior was indexed by the frequency of time-stamped player contact events detected on video review. Contact was defined as any within-match instance of physical contact to the player's head/neck or body by another player and/or object. Contact events were further characterized by skill execution, ball control of the contacted player, the phase of play, contact descriptor, contact outcome and umpire-determined legality of contact (see Table 1).

**Table 1.** Characterization factors for video contact events.

Phase of play	Ball control
Offence (team in control) Defence (opponent team in control) Contested Play (ball in dispute)	In possession Not in possession Other (e.g. stoppage)
**Skill execution**	**Contact descriptor**
Marking Handballing Receiving handball Tackling opponent Kicking the ball Contested ball on the ground Running or standing with the ball Bumping or blocking (shepherding) Running or standing without the ball Intercept mark Smothering Knocking the football on Spoiling	Collision during marking contest Being tackled by opponent Tackling opponent Bumping or blocking (shepherding) opponent Contested ball Player being bumped or blocked (shepherded) Other contest Player contact with ground or struck by ball Player contact with post or fence
**Contact outcome**	**Contact legality**
No Injury Player with visible discomfort Player requiring medical aid Player removed from match	Illegal contact with free kick awarded Illegal contact with no free kick awarded Contact was not illegal

Subsequently, all timestamped X-Patch^®^ output underwent verification per player per device by attempting to precisely match X-Patch detected HAEs to video-detected contact events using a window of +/- one minute to account for potential discrepancies between the estimated timestamp provided by the X-Patch and exact video timestamps [28,35,40].

#### Injury surveillance

Injury surveillance was conducted by three research personnel stationed around the arena. Prior to data collection, research personnel received training in the recognition of concussion, and use of a standardised injury report form to document player details (e.g., guernsey number, quarter, HG or no HG match half), injury details (e.g., did the player leave the playing surface with an injury), and injury categories derived from The Australian Sports Injury Dictionary [41] including injury descriptors (e.g., fall, tackle), injury pathology (e.g., open wound, fracture), and the injured body region (see Appendix A for complete list). Three injury outcomes (suspected SRC, non-SRC head injury, body region injuries) were based on data collected using the operational injury definitions (see Table 2).

**Table 2.** Definitions of suspected SRC, non-SRC head injury and other body region injuries.

Injury type/variable	Definition	Measure
Suspected SRC	In accordance with the recent CISG consensus statement, concussion was defined as a traumatic brain injury induced when a biomechanical force is sustained to the head, neck, face or elsewhere on the body and transmitted to the brain. SRC may or may not involve loss of consciousness, cannot be accounted for by other injuries, medication or medical and psychological factors, and typically involves transient neurological impairment that resolves spontaneously over time	Observed signs and/or reported symptoms of a SRC, and headgear use at the time of impact were recorded in accordance with the HIA form
Non-SRC head injury	Non-SRC head injuries were defined as those that occurred to the head, superficial to the skull and excluding SRC (e.g., bruising, abrasions)	Observed signs and symptoms were recorded along with body region, pathology, impact descriptor and headgear use at the time of impact using categories derived from The Australian Sports Injury Data Dictionary
Injury to other body regions	Other body region injury rates were defined as injuries sustained to any part of the body, excluding SRC and non-SRC head injuries	Observed signs and symptoms were recorded with body region, pathology, impact descriptor and headgear use at the time of impact using categories derived from The Australian Sports Injury Data Dictionary

CISG: Concussion in Sport Group; HIA: Head injury assessment form; SRC: Sport-related concussion.

Events were coded as a *match incident* for any within-match physical complaint reported or displayed by a player where the player was observed to exhibit signs of pain after an observed impact (i.e., both the incident and effect of trauma had to be observed); a *medical assessment* injury if a clinician (doctor, paramedic or physiotherapist) was consulted or provided treatment of an injury during or after the match; or a *missed match* injury if a player missed a subsequent match(es) due to the injury. Research personnel recorded injury information on match day. They subsequently conducted a follow-up phone call with coaches, players and parents during the week following the match to confirm details of any injury-related medical assessments occurring post-match in either physician's rooms or in an emergency department, as well as to record whether or not injuries resulted in missed matches (See Appendix B for record sheet).

Suspected SRC were identified using a previously employed method screening SRC in community football [42]. The AFL Community Head Injury Assessment (HIA) form was used to record signs and symptoms of SRC (See Appendix C). This tool was based on the elite AFL rapid sideline HIA form where SRC injuries were detected with 89% sensitivity (95% CI: 75.44–96.21%) and 98% specificity (95% CI: 92.03–99.72%) when completed by sports medicine physicians [43]. The term “suspected SRC” was chosen as research personnel were not medically trained to confirm a SRC diagnosis, nor had confirmation of a SRC diagnosis post-medical assessment, if any.

### Analysis

Measures of inter-and intra-rater consistency were conducted for all descriptors during video review using Cohen's Kappa, intraclass correlation (ICCs) and percentage agreement. Continuous normally distributed variables (age, weight, height, frequency of contact events) were summarized using means (standard deviations) and compared with Student's *t*-test. Nominal and ordinal variables were summarised using proportions and compared using chi-square tests. The sum of video-detected player contact events divided by the total number of player participations (where one participation is one player present at one match) was calculated in order to provide the rate of contact events per player per match with corresponding 95% confidence intervals (*CIs*).

$$Rate\;of\;contact\;events=\frac{\sum Player\;contact\;events}{\sum \;Player\;participations}$$

Statistical significance was defined as p < 0.05 with Bonferroni correction where appropriate. All data were analysed with SPSS (IBM version 26).

## Results

**Figure 2.**
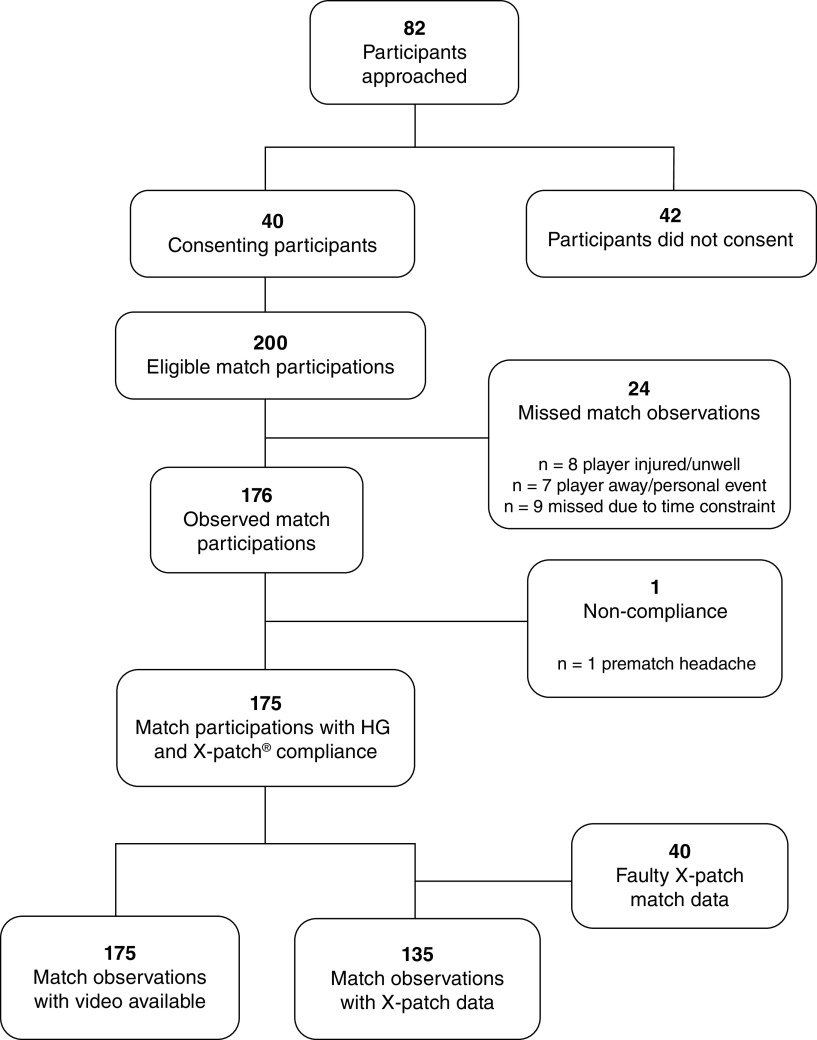
Participant and data flowchart.

Across the four teams approached there were n = 82 players eligible to participate in the study. While the U14 male team coach and club personnel agreed to participate, no individual U14 player assented to the trial and this team (n = 23) therefore did not participate in data collection. Of the total of n = 59 players from the three participating teams, n = 40 (68%) players provided consent to participate (see Figure 2), with an average age of 12.43 (SD = 1.36) years. In total, there were 176 player participations across the 15 matches observed.

Table 3 outlines demographics by sex, indicating that male participants were younger, shorter and lighter than female participants.

**Table 3.** Player demographics.

	Male	Female
n (%)	15 (38%)	25 (62%)
Age (years), mean (SD)	11.40 (1.06)	13.04 (1.14)
Height (cm), mean (SD)	154.00 (5.24)	160.60 (6.71)
Weight (kg), mean (SD)	44.15 (7.66)	52.11 (8.53)

### Compliance & feasibility

Across the 15 matches, 99% HG compliance and 99% X-Patch compliance was observed (i.e., only one player in one match declined wearing HG and X-Patch due to a reported headache on match day). Some players requested sporting tape to be placed on the Velcro chin strap to limit skin abrasion from HG straps. No X-Patch device fell off during the match.

### Video review

A total of 3061 player contact events were detected during video review, of which 3% (n = 90) were coded as head/neck contact, 96.5% (n = 2955) were coded as body contact, and 0.5% (n = 16) were coded as unknown due to a restricted camera angle preventing determination of site of contact. Analysis of rating consistency for descriptors used to characterize player contact events revealed good to excellent inter-rater (ICC: 0.616–.842) and intra-rater (ICC: 0.722–1.00) reliability (see Appendix D).

There was no difference in the frequency of head/neck contact with HG (n = 46, 51.1%) or no HG (n = 44, 48.9%), p = .916.

There were no significant differences found in head/neck contact events with HG or no HG for ball control, p = 0.608, phase of play, p = 0.292, skill execution, p = 0.291, contact descriptor, p = 0.649. outcome, p = 0.831 or illegal contact, p = 0.642 (See Appendix E). A total of n = 15 (16.6%) head/neck contact events were associated with illegal contact.

There was no difference in the frequency of body contact events with HG (n = 1473, 49.8%) or no HG (n = 1482, 50.2%), p = 0.883. When comparing by contact descriptor, the frequency of body contact with no HG (n = 384) was significantly higher than with HG (n = 307) during a contested ball situation, p < 0.001. There were no significant differences with HG or no HG in body contact for ball control, p = 0.383, phase of play, p = 0.431, skill execution, p = 0.361, outcome, p = 0.435 or illegal contact, p = 0.570 (see Appendix E).

#### Between-subject differences

There was no difference between male and female players in the frequency of head/neck contact events. Body contact events were higher among females in the no HG condition (see Table 4).

**Table 4.** Mean and standard deviations of player contact rates per player per match.

	All Impacts		With HG		No HG	
Sex	Head	Body	Head	Body	Head	Body
Male	0.43 (0.48)	13.38 (9.35)	0.21 (0.27)	7.21 (4.73)	0.22 (0.27)	6.17 (5.13)
Female	0.56 (0.41)	19.65 (8.56)	0.28 (0.33)	9.32 (4.44)	0.28 (0.27)	10.33 (4.91)
p-value	0.36	0.037	0.52	0.16	0.46	0.015

p-value significance: <0.05.

HG: Headgear.

#### Within-subject differences

Among female players (n = 25), there were comparable frequencies in contact events with and without HG for head/neck contact, p = 0.896, and body contact, p = 0.050 (see Table 5). When exploring differences by contact descriptor among body contact events in female players, however, frequency of body contact events was significantly greater during contested ball situations with no HG (n = 299) in comparison to contact events with HG (n = 196), p < 0.001. Conversely, frequency of body contact events during tackling in female players were significantly greater with HG (n = 247) relative to body contact events with no HG (n = 198), p < 0.001. Upon closer inspection of the data it appeared that this effect was, in fact, largely driven by the same group of female players (n = 11) who sustained frequent body contact events under HG and no HG conditions. Frequency of body contact events with HG or no HG in female players did not differ across ball control, p = 0.030 (not significant after Bonferroni correction), phase of play, p = 0.068, skill execution, p = 0.341, outcome, p = 0.105, or illegal contact, p = 0.691.

**Table 5.** Frequency of head/neck and body contact events per player by sex and headgear condition.

	Female players (n = 25)		Male players (n = 15)	
Condition	Head/neck contact events (n = 58), n (%)	Body contact events (n = 1974), n (%)	Head/neck contact events (n = 32), n (%)	Body contact events (n = 981), n (%)
Headgear	30 (51.7%)	943 (47.8%)	16 (50.0%)	530 (54.0%)
No headgear	28 (48.3%)	1031 (52.2%)	16 (50.0%)	451 (46.0%)
p-value	0.896	0.050	0.876	0.013

Among male players (n = 15), frequency of head/neck contact events with and without HG were comparable, p = 0.876 (see Table 5). Body contact events with HG were, however, significantly more frequent than body contact events with no HG among male players, p = 0.013. There were no differences in frequency of body contact events with or without HG among male players across ball control, p = 0.255, phase of play, p = 0.423, skill execution, p = 0.475, contact descriptor, p = 0.488, outcome, p = 0.105, or illegal contact, 0.691.

### Association between risk taking & video detected contact events

For those who completed the modified RPS (n = 25), there was no statistically significant association between modified RPS scores and body, n = 1967, Spearman's rho = -0.227, p = 0.275, or head/neck contact events, n = 60, Spearman's rho = -0.322, p = 0.117.

### Injury surveillance outcomes

There were 11 match incidents detected during the live injury surveillance among n = 10 players (n = 5 male players), with n = 6 of these incidents occurring with HG (see Table 6). Two incidents occurring with HG were suspected SRCs (n = 1 male player). During the video review, it was shown that n = 3 (27%) match incidents were due to the player being bumped by the opposition, n = 2 (18%) match incidents were due to the player being tackled, n = 2 (18%) match incidents were due contact during a contested ball on the ground while others were due to contact during a marking contest (n = 1, 9%), contact with the ground (n = 1, 9%) or other contest (n = 1, 9%), while n = 1 (9%) match incident (occurring in match 6) was not visible during the video review due to a restricted camera angle. No match incidents caused a player to miss the subsequent match, and none were medically assessed, including suspected SRCs. For one suspected SRC (match 13), the female player sustained a head impact after being bumped/blocked by an opposition player while attempting to gain possession of the ball and remained on the field. Upon follow-up post-match, the player reported having a headache and the player's parent noted the player was ‘more emotional than usual’. For the other suspected SRC (match 15), the male player sustained a head impact after being tackled by an opposition player while running with possession of the ball. The player experienced a blood nose and reported having a very brief period of ‘dizziness’ post-impact. The player was removed from the field but returned to play in the next quarter.

**Table 6.** Match incident details.

Match	Quarter	Sex	HG Use	Left the playing surface?	Returned to playing surface?	Injury type
1	4	Female	No	Yes	Yes	Superficial
1	3	Female	No	Yes	Yes	Superficial
4	3	Female	Yes	Yes	Yes	Superficial
6	4	Male	No	No	–	Superficial
7	2	Male	No	No	–	Superficial
10	2	Female	Yes	Yes	Yes	Superficial
13	1	Female	Yes	No	–	Suspected concussion
14	3	Male	Yes	No	–	Superficial
14	2	Male	No	No	–	Sprain or strain
14	4	Male	Yes	No	–	Superficial
15	3	Male	Yes	Yes	Yes	Suspected concussion

Superficial injury includes grazes, bruises or blisters.

HG: Headgear.

### Accelerometer review

Due to a software product error in the synchrony of timestamps for X-Patch^®^ data processed with IMS, correctly timestamped X-Patch data was only available for n = 135 participations (i.e., 76% of the total 176 participations) accounting for 79.3% (n = 2426) of the total n = 3061 player contacts detected on video. Of these n = 2426 video-detected contacts, n = 69 (2.8%) were coded as head impacts of which n = 24 (34.8%) were matched with a HAE ≥10 *g*, and n = 5 (7.2%) were matched with a HAE ≥30 *g*. Moreover, of n = 2426 video-detected contacts, n = 2345 (96.7%) were coded as body impacts of which n = 470 (19.4%) were matched with a HAE ≥10 *g*, and n = 51 (2.2%) were matched with a HAE ≥30 *g*. Overall, only 20% of video-detected player contact events were matched with X-Patch detected HAE ≥10 *g*.

## Discussion

In this novel pilot study evaluating player behavior across HG and no HG conditions in junior Australian football, HG was generally well tolerated and HG compliance was excellent. Nevertheless, the compliance may be artificially biased as one entire U14 boys Division 1 team declined voluntary consent. While the club nominated this team, the coach was in agreeance, and some parents consented, there appeared to be a collective decision among the players to decline consent. Specific reasons behind this decision were unable to be explored, however survey studies regarding barriers to HG compliance suggest that factors such as dislike of HG and discomfort (e.g., it causing players to feel too hot) are associated with HG reluctance [20]. Of the remaining three teams, 68% of players agreed to participate, which is a reasonable proportion based upon previous similar work [2].

Overall, head/neck contact events were substantially less frequent than body contact events, indicating that the rate of head/neck contact was reasonably well governed and disincentivized by the Laws of Australian Football and umpires adjudicating the observed matches. Given the sample mean age of 12 years, it is likely that this is at least in part associated with the modified rules that apply to the younger age groups such as limited shepherding, bumping, and modified tackling [31], with only 16% of head/neck contact events being associated with illegal contact.

Frequency of head/neck contact events across HG and no HG conditions was not statistically different and, in fact, was remarkably similar for both male and female players. As this was an exploratory pilot study, an analysis of game situation and context was included which indicated that there was no significant difference in head/neck contact events with HG or no HG across situations of ball control, phase of play, skill execution, contact descriptor, outcome or illegal contact situations. This is consistent with the findings from the aforementioned recent systematic review which demonstrated little evidence for reduced risk of non-SRC head injury (i.e., bruises, grazes sustained superficial to the skull) or SRC with HG [8], and also a previous injury surveillance cohort study by our group [9] in which 10 mandated HG and 10 no HG junior teams were followed across two Australian football seasons and injuries (suspected SRC, non-SRC head injury and other body region injuries) were recorded by primary data collectors. There was very little evidence that HG mitigated SRC or non-SRC head injury risk. The above-mentioned study informed the design of the current crossover design methodology with the added advantages that players served as their own HG and no HG control, thereby controlling for individual player factors (e.g., level of aggression, risk propensity, safety attitudes), level of the competition, and match day factors (e.g., weather, match number within season).

Regarding body contact events, there were no significant differences with HG or no HG in body contact across situations of ball control, phase of play, skill execution, outcome or illegal contact. For contact descriptor however, there were more frequent body contacts in the no HG condition in contested ball situations. This appeared to be largely driven by females, as female players had a significantly greater frequency of body contacts in the no HG condition per player per match than male players, particularly in contested ball situations. By contrast, male players sustained more body contacts with HG across playing situations.

The concept of risk compensation in sport has largely been explored in predominantly male cohorts [2,20,44], and proposes that if a person perceives an intervention (e.g., HG use) to have lowered their level of injury risk, they may lower their inhibitions and play more aggressively, thus returning themselves to the original level of risk. Concern has been expressed that the use of HG may secondarily result in this phenomenon, and that players might actually be at increased risk of sustaining SRC and non-concussive injuries to the rest of the body compared with their non-HG using counterparts in soccer, rugby and Australian football [45–47]. We observed very weak evidence in support of risk compensation and HG wearing in the present study, with an approximately equal proportion of match incidents across HG and no HG conditions, some indication of more body contact events with HG than without HG in males only, and no significant difference across HG conditions for head/neck contact events.

Given that just over half of the participants completed the modified risk propensity scale, we were unable to examine sex differences and association with HG, however there was no correlation between frequency of head/neck or body contacts and risk propensity for the overall sample. Other more comprehensive risk taking scales [e.g., the Domain-Specific Risk Taking Measure; 48] may have enabled a more in-depth analysis of this concept, however such scales are not designed for use with paediatric cohorts. Certainly sex differences in safety behavior and risk taking in contact sports such as Australian football requires greater investigation given the recent proliferation in female participation [6].

## Limitations

The methodological and resource challenges associated with undertaking gold-standard injury surveillance studies in junior community football cohorts are well documented [23]. Findings from a recent junior cohort HG study by our group indicated that in order to design a RCT with sufficient numbers to investigate the association between HG use and the risk of sustaining an injury resulting in medical attention or a subsequent missed match, more than 2500 players participating across a season, with match exposure of approximately 30,000 hours would be required [8]. Due to the relatively low frequency of these types of injuries in junior football cohorts, as a preliminary pilot investigation the current study attempted to be the first to quantify the frequency of head/neck and body contacts with and without HG as identified on video review as a marker of player behavior in a crossover, controlled pilot trial. Inter-rater reliability of video coded contact events was very good. With only n = 90 head/neck contact events recorded, it is still possible that the current study was insufficiently powered to detect differences across the HG conditions. Additionally, data were collected from only one club for whom the issue of SRC is a real priority, and so it cannot be assumed that the findings of this pilot study would necessarily extrapolate to other community junior football clubs. Future studies need to involve a large, national sample, including both male and female athletes across age ranges representative of participation. The injuries documented in this study were observed by research personnel with varying levels of training and familiarity of the measures used, and none were qualified medical physicians or certified athletic trainers. While this is not ideal, objective data collection by these means has been shown to be superior to retrospective player self-report of injuries [23].

A secondary aim of quantifying both the frequency and magnitude of HAEs as a marker of injury risk was unable to be achieved given the poor reliability of the X-Patch accelerometer device. More recently, the X-patch device has been found by others to have questionable reliability, particular for measurement of PRA [34,49,50] and is no longer commercially available. Nonetheless, as a pilot study investigating feasibility players were compliant with the use of the accelerometer device, and it was well tolerated, paving the way for future trials in junior cohorts if more reliable devices can be developed.

## Conclusion

This novel HG case-crossover pilot study, the first of its kind in junior Australian football including both male and female players, found no difference in rate of head/neck contact events across HG conditions. Females had a significantly greater frequency of body contacts in the no HG condition than male players, particularly in contested ball situations. Males sustained more body contacts with HG than without HG across playing situations. Injury rate *per se* was not associated with HG use, although the study was significantly underpowered to detect small differences. These findings suggest that the use of HG in junior football is not associated with rate of head contact, and that the association between HG use and body contact may differ across sexes.

The current pilot has demonstrated that it is feasible to trial HG use in junior Australian football players, and utilise measures such as rate of contact identified in video review to examine player behavior across HG conditions to investigate possible aspects of risk compensation associated with HG use. Future trials examining the effectiveness of soft-shell padded HG for SRC prevention in junior Australian football, undertaken with HG models demonstrating sufficient impact attenuation properties developed and manufactured according to Australian standards, should aim to concurrently evaluate player behavior as a marker of risk compensation. In order to sufficiently power such studies, cooperation with junior football leagues would likely be required as individual consenting teams or even clubs would not yield sufficient numbers for true injury surveillance. Given the rapid increase in female participation numbers and some indication from the current study that the association between player behavior and HG use may differ by sex, the inclusion of female players going forward will be paramount.

## Future perspective

There is a need to develop commercially available HG models that meet required standards and demonstrate sufficient impact attenuation in both the laboratory and in the field. In addition, randomised controlled trials of HG need to be adequately powered to provide a robust determination of association with injury risk, with inclusion of female collision sports athletes. Pending evidence in support of future HG development for SRC prevention, barriers to uptake in junior collision sports would additionally need to be identified and addressed through education and promotion of a safety culture. In junior community football, educational focus on concussion management in recognise and remove from play protocols is a priority.

**Summary points**

- Evidence for soft-shell padded headgear (HG) use in prevention of sport-related concussion remains limited.
- HG studies conducted in rugby and soccer, with predominantly male players, are not readily translatable to inform the use of HG in junior male and female Australian football cohorts.
- Despite this, HG is inconsistently mandated as a preventive strategy for sports-related concussion (SRC) in junior and youth Australian football.
- In contact sport, the risk compensation theory posits that players feel that they can tackle harder and are more confident when wearing HG which may result in more aggressive play, with less concern for safety.
- In this case crossover pilot, consistent with previous findings, there was little evidence that HG mitigated SRC, or non-SRC head injury, risk in junior Australian football.
- Frequency of head/neck contact events across HG and no HG conditions was not statistically different suggesting little indication of risk compensation, and any association between HG use and body contact events may differ across the sexes.
- Future RCTs examining the effectiveness of soft-shell padded HG for SRC prevention in junior Australian football, undertaken with HG models demonstrating sufficient impact attenuation properties developed and manufactured according to Australian standards, should aim to concurrently evaluate player behavior as a marker of risk compensation.
