# Supplementary material for: A pilot case crossover study of the use of padded headgear in junior Australian football
Source: Concussion. 2023 Jan 17;7(4):CNC99. doi: 10.2217/cnc-2022-0005 (PMC9855304; doi:10.2217/cnc-2022-0005)
Supplement: Supplementary file 1 [file cnc-07-99-s1.docx]

| **Appendix A Table**  Contents of the Standardized Injury Report Form | | | |
| --- | --- | --- | --- |
| **Player Details** | | | |
| Date of injury Guernsey number  Player name Time (quarter) of injury  Sex Sporting field that injury took place | | | |
| **Injury Details** | | | |
| Did the player leave the field with an injury?  Did the player return to the field after the injury?  Was this a recurring injury? (i.e., same injury at the same site)?  Was the player wearing HG?  Has the injury been verified by a health professional? | | | |
| **Injury Descriptor** | | **Injury Pathology** | |
| Fall  Collision with other player/object  Ball contest  Tackled  Over exertion  Accelerating/Decelerating | | Superficial (includes bruise, blister, graze)  Open wound  Fracture  Dislocation  Sprain or strain  Injury to internal organ | |
| **Body Region List** | | | |
| Head  Face  Eye  Neck  Chest  Abdomen  Back | Pelvis  Shoulder  Upper Arm  Elbow  Forearm  Wrist  Hand | | Hip  Thigh  Knee  Lower leg  Ankle  Foot |
|  | | | |

**Appendix B**

**Missed Game Record**

*This form should only filled out for players who have missed a game because of an injury sustained in a previous football game*

**MISSED GAMES BECAUSE OF AN INJURY SUSTAINED IN A PREVIOUS GAME MUST BE RECORDED**

1. **Protocol**

In this study, the ***severity of injury*** is measured by ***number of games missed***.

E.g. If a player has missed three games due to a concussion that you have recorded in a previous game, that would indicate that the participant is experiencing ongoing symptoms and cannot play.

We want to capture this data to see whether injuries are more severe for those who wear vs. those who don’t wear headgear.

Please continue to liaise with PDC, parents, coaches, team managers, etc. to follow-up with any injuries that may result in missed games.

**IF A PLAYER SUSTAINS AN INJURY THAT LOOKS SEVERE, BE SURE TO RECORD IF THEY DO NOT PLAY THE FOLLOWING WEEK DUE TO THAT INJURY BY FILLING OUT THE INFORMATION BELOW.**

1. **Missed Game Record Form**

| Did the child miss the game due to an injury sustained in a previous game? *If YES*, what was the date of the injury? |  |
| --- | --- |
| Who did you confirm this with (E.g. PDC, team manager, parent, player)? |  |
| How did you confirm this information (E.g. email, phone call, at the game)? |  |

| Player name: |  |
| --- | --- |
| Guernsey number: |  |
| Age group (e.g. U11s): |  |
| Gender: |  |
| Injury type: |  |

**Appendix C**

**COMMUNITY FOOTBALL HEAD INJURY ASSESSMENT**

1. **GENERAL INFORMATION**

Player Name: **** Club: ****

Examiner Name: **** Date: ****

Quarter: **** Approximate Time in Quarter: ****

1. **STRUCTURAL HEAD OR NECK INJURY**
2. Are there clinical features of a serious or structural head and/or neck injury  **Yes**  No

requiring urgent and emergency hospital transfer?^1^

1. **REMOVAL FROM PLAY**

The player **must** be removed from play with **any** of the following clinical features^2^ observed directly, reported by others or from video review (if available):

|  |  | **YES** |  | **NO** |
| --- | --- | --- | --- | --- |
|  | Observed Directly | Reported | Video Review |  |
| 2. Loss of consciousness or prolonged immobility (> 2 seconds) |  |  |  |  |
| 3. No protective action in fall to ground (not bracing for impact) |  |  |  |  |
| 4. Impact seizure (stiffening arms or legs on impact) |  |  |  |  |
| 5. Balance disturbance (loss of control over movements) |  |  |  |  |
| 6. Dazed, blank/vacant stare or not their normal selves |  |  |  |  |
| 7. Unusual behaviour change for the player |  |  |  |  |
| 8. Confusion or disorientation |  |  |  |  |
| 9. Memory impairment (e.g. fails Maddocks questions^1^) |  |  |  |  |
| 10. Player reports concussion symptoms^1^ |  |  |  |  |

^1^ Refer to the AFL Concussion Management Guidelines available on the AFL Community website: [http://www.aflcommunityclub.com.au/](http://www.aflcommunityclub.com.au/index.php?id=66).

^2^ Example videos of each clinical feature are available on the AFL Community website.

1. **OUTCOME AND ACTION**

If ‘Yes’ is selected for question 1, it requires an ambulance to be called for immediate transfer to hospital

If ‘Yes’ is selected for questions 2-9, it requires immediate removal from play and medical assessment^3^

If ‘No’ is selected for questions 1-10, no criteria for removal from play for concussion^4^

^5^ A player who is removed from play for concussion or possible concussion must not return to play until cleared by a doctor.

^4^ A player cleared to play requires regular checks at least every 30 minutes and removal from play with any deterioration.

1. **SIGNATURE OF EXAMINER**

Signed: ** Date: ** Time completed: **

**F. MEDICAL CLEARANCE – TO BE COMPLETED BY A MEDICAL PRACTITIONER**

I have examined: **** following the above head injury and declared him/her medically fit^5^ to train and play.

Practitioner Name: **** Medical Practice Stamp:

Signed: ****

Date: ****

^5^ Please refer to the medical check list over the page when assessing the player and determining his medical fitness to train and play.

**NOTES FOR THE EXAMINING MEDICAL PRACTITIONER**

Please refer to the AFL Concussion Management Guidelines available via the following website:

[http://www.aflcommunityclub.com.au/](http://www.aflcommunityclub.com.au/index.php?id=66)

A concussed footballer requires a medical clearance to return to training or competition.

In accordance with the current Concussion Guidelines, there is no mandatory period of time that an Australian Football player must be withheld from play following a concussion. The duration of exclusion from play is based on an individual’s recovery as managed by a medical practitioner. It would not be unreasonable to clear the player to return to structured training with a second consultation to clear the player for full training/match play.

The minimum standard is that a player must be symptom free at rest and with exertion, determined to have returned to baseline level of cognitive performance, and is confident and comfortable to return to play.

Screening computerised cognitive tests provide a practical method for the assessment of cognitive recovery. A number of screening computerised cognitive test batteries have been validated for use following concussion in sport and are readily available (e.g. CogState Sport, ImPACT). Conventional imaging (e.g. CT or MRI) should be considered in cases where there is concern regarding an underlying structural injury.

The following is a guide to the medical examination of a concussed player:

- Are there any neurological symptoms on questioning or signs on examination?
- Is the player experiencing ongoing symptoms suggestive of concussion?
- Does the player experience concussion type symptoms when undertaking physical activity?
- Has the player not returned to their usual work or education?

If the answer to any of the above questions is ‘Yes’, the player requires further observation or a referral for specialist assessment.

If the player clears the above tests, ensure as per the AFL Community Concussion Guidelines the player complies with a graduated return to train and play protocol, with instructions for further medical assessment if the symptoms return.

Difficult or complicated cases (e.g. prolonged recovery or recurrent concussion) should be referred to a clinician or neurologist with expertise in concussion.

**Appendix D**

Inter-rater reliability was conducted for 15 randomly selected video events while intra-rater reliability was conducted for an additional 30 randomly selected video events previously coded by JR and JN (15 impacts each). Both raters coded these cases independently. Cohen’s Kappa was calculated for nominal factors and intraclass correlations (ICCs) were calculated for ordinal factors [45, 46]. Percentage agreement was also calculated, and produced as the number of agreement scores divided by the total number of scores. Average measures intraclass correlations (ICCs) were based on absolute agreement using a two-way mixed effects model with coders as the fixed effect and provided with 95% confidence intervals. Cohen’s Kappa revealed mostly *moderate* to *very good* inter-and intra-rater reliability [47]. These were consistent with the ICCs, which revealed mostly *good* to *excellent* reliability and percentage agreement with *moderate* to *almost perfect* agreement [48, 49]. Body Impact Location had *fair* inter-rater reliability (Kappa = .324), however percentage agreement and ICCs revealed *moderate* (67%) and *good* (.616) agreement respectively.

| **Appendix D Table**  Interrater Reliability Provided by Intra-Class Correlations with 95% Confidence Intervals, Cohen’s Kappa and Percentage Agreement | | | | | | | | | |
| --- | --- | --- | --- | --- | --- | --- | --- | --- | --- |
| Field | IRR1 | | | IRR2 | | | IRR3 | | |
|  | ICC (95% CI) | K | % | ICC (95% CI) | K | % | ICC (95% CI) | K | % |
| Ball Control | .788 (.386 - .928) | .634 | 93 | 1.00 | 1.00 | 100 | .920 (.767 - .973) | .842 | 93 |
| Phase of Play | .731 (.206 - .909) | .643 | 80 | .806 (.429 - .934) | .625 | 80 | 1.00 | 1.00 | 100 |
| Skill Execution | .842 (.486 - .949) | .579 | 67 | .964 (.893 - .988) | .826 | 87 | .824 (.470 - .941) | .832 | 87 |
| Impact Descriptor | .676 (.039 - .891) | .516 | 60 | .722 (.201 - .905) | .755 | 80 | .805 (.422 - .934) | .803 | 87 |
| Outcome | 1.00 | 1.00 | 100 | 1.00 | 1.00 | 100 | 1.00 | 1.00 | 100 |
| Illegal Contact | 1.00 | 1.00 | 100 | 1.00 | 1.00 | 100 | 1.00 | 1.00 | 100 |
| Body Impact Location | .616 (-.156 - .872) | .324 | 67 | .939 (.824 - .979) | .643 | 93 | .920 (.767 - .973) | .842 | 93 |
| Head Impact location | .933 (.807 - .977) | .867 | 93 | 1.00 | 1.00 | 100 | 1.00 | 1.00 | 100 |
| IRR1 = Interrater Reliability  IRR2 = Intra-rater reliability for JR  IRR3 = Intra-rater reliability for JN  ICC = Intraclass correlations  K = Cohen’s Kappa | | | | | | | | | |

| **Appendix E Table**  Distribution of Video-Detected Contact Events Per Headgear (HG) Condition | | | | | | |
| --- | --- | --- | --- | --- | --- | --- |
|  | **Head/Neck Contact Events**  N = 90 | | | **Body Contact Events**  N = 2955 | | |
|  | No HG  n = 44 | HG  n = 46 | *p* | No HG  n = 1482 | HG  n = 1473 | *p* |
| **Ball Control** |  |  |  |  |  |  |
| In possession | 14 (31.8%) | 17 (37.0%) | .608 | 342 (23.1%) | 370 (25.1%) | .383 |
| Not in possession | 30 (68.2%) | 29 (63.0%) |  | 1138 (76.8%) | 1100 (74.7%) |  |
| Other (stoppage, fight) | 0 | 0 |  | 2 (0.1%) | 3 (0.2%) |  |
| **Phase of Play** |  |  |  |  |  |  |
| Offence (team in possession) | 14 (31.8%) | 13 (41.3%) | .292 | 370 (25.0%) | 381 (25.9%) | .431 |
| Defence (opposing team in possession) | 3 (6.8%) | 6 (13.0%) |  | 366 (24.7%) | 345 (23.4%) |  |
| Contested play | 27 (61.4%) | 21 (45.7%) |  | 746 (50.3%) | 745 (50.6%) |  |
| Unknown/Unclear | 0 (0.0%) | 0 (0.0%) |  | 0 (0.0%) | 2 (0.1%) |  |
| **Skill Execution** |  |  |  |  |  |  |
| Marking | 7 (15.9%) | 6 (13.0%) | .291 | 71 (4.8%) | 70 (4.8%) | .361 |
| Handballing | 8 (18.2%) | 1 (2.2%) |  | 61 (4.1%) | 56 (3.8%) |  |
| Receiving handball | 0 (0.0%) | 1 (2.2%) |  | 18 (1.2%) | 21 (1.4%) |  |
| Tackling opponent | 3 (6.8%) | 4 (8.7%) |  | 358 (24.2%) | 342 (23.2%) |  |
| Kicking the ball | 2 (4.5%) | 4 (8.7%) |  | 103 (7.0%) | 133 (9.0%) |  |
| Contested ball on the ground | 13 (29.5%) | 18 (39.1%) |  | 488 (32.9%) | 460 (31.2%) |  |
| Running or standing with the ball | 5 (11.4%) | 8 (17.4%) |  | 155 (10.5%) | 144 (9.8%) |  |
| Bumping or blocking (shepherding) | 1 (2.3%) | 1 (2.2%) |  | 130 (8.8%) | 142 (9.6%) |  |
| Running or standing without the ball | 0 (0.0%) | 0 (0.0%) |  | 40 (2.7%) | 30 (2.0%) |  |
| Intercepting | 0 (0.0%) | 0 (0.0%) |  | 1 (0.1%) | 0 (0.0%) |  |
| Smothering | 0 (0.0%) | 0 (0.0%) |  | 8 (0.5%) | 11 (0.7%) |  |
| Knocking the football | 4 (9.1%) | 1 (2.2%) |  | 41 (2.8%) | 58 (3.9%) |  |
| Spoiling | 0 (0.0%) | 0 (0.0%) |  | 2 (0.1%) | 0 (0.0%) |  |
| Unknown/Unclear | 1 (2.3%) | 2 (4.3%) |  | 6 (0.4%) | 6 (0.4%) |  |
| **Contact Descriptor** |  |  |  |  |  |  |
| Collision during marking contest | 7 (15.9%) | 5 (10.9%) | .649 | 59 (4.0%) | 54 (3.7%) | .013 |
| Being tackled by opponent | 10 (22.7%) | 13 (28.3%) |  | 310 (20.9%) | 356 (24.2%) |  |
| Tackling opponent | 2 (4.5%) | 5 (10.9%) |  | 356 (24.0%) | 332 (22.5%) |  |
| Bumping or blocking (shepherding) | 0 (0.0%) | 1 (2.2%) |  | 115 (7.8%) | 140 (9.5%) |  |
| Contested ball^a^ | 13 (29.5%) | 11 (23.9%) |  | 384 (25.9%) | 307 (20.8%) |  |
| Player being bumped or blocked (shepherded) | 5 (11.4%) | 5 (10.9%) |  | 158 (10.7%) | 157 (10.7%) |  |
| Other contest | 5 (11.4%) | 2 (4.3%) |  | 58 (3.9%) | 70 (4.8%) |  |
| Player contact with ground or struck by ball | 2 (4.5%) | 4 (8.7%) |  | 42 (2.8%) | 56 (3.8%) |  |
| Player contact with post or fence | 0 (0.0%) | 0 (0.0%) |  | 0 (0.0) | 0 (0.0) |  |
| Unknown/Unclear | 0 (0.0%) | 0 (0.0%) |  | 0 (0.0) | 1 (0.1%) |  |
| **Outcome** |  |  |  |  |  |  |
| No injury | 38 (86.4%) | 39 (84.8%) | .831 | 1452 (98.0%) | 1452 (98.6%) | .435 |
| Player with visible discomfort | 6 (13.6%) | 7 (15.2%) |  | 24 (1.6%) | 16 (1.1%) |  |
| Player requiring medical aid | 0 (0.0%) | 0 (0.0%) |  | 0 (0.0%) | 0 (0.0%) |  |
| Player removed from match | 0 (0.0%) | 0 (0.0%) |  | 0 (0.0%) | 0 (0.0%) |  |
| Unknown/Unclear | 0 (0.0%) | 0 (0.0%) |  | 6 (0.4%) | 5 (0.3%) |  |
| **Illegal Contact** |  |  |  |  |  |  |
| Illegal contact with free kick awarded | 1 (2.3%) | 2 (4.3%) | .642 | 5 (0.3%) | 8 (0.5%) | .570 |
| Illegal contact with no free kick awarded | 7 (15.9%) | 5 (10.9%) |  | 20 (1.3%) | 17 (1.2%) |  |
| Contact was not illegal | 36 (81.8%) | 38 (82.6%) |  | 1454 (98.1%) | 1446 (98.2%) |  |
| Unknown/Unclear | 0 (0.0%) | 1 (2.2%) |  | 3 (0.2%) | 2 (0.2%) |  |
| **^a^** The number of body impacts during a contested ball was significantly different with and without HG after FWE Bonferroni corrections where p = 0.05 / 18 is p = .003. | | | | | | |
